# Stent appearance in a novel silicon-based photon-counting CT prototype: *ex vivo* phantom study in head-to-head comparison with conventional energy-integrating CT

**DOI:** 10.1186/s41747-023-00333-0

**Published:** 2023-04-25

**Authors:** Emma Verelst, Nico Buls, Johan De Mey, Koenraad Hans Nieboer, Frans Vandenbergh, Dominic Crotty, Paul Deak, Albert Sundvall, Staffan Holmin, Aron De Smet, Steven Provyn, Gert Van Gompel

**Affiliations:** 1 Department of Radiology, Vrije Universiteit Brussel (VUB), Universitair Ziekenhuis Brussel (UZB), Laarbeeklaan 101, 1090 Brussels, Belgium; 2 GE Healthcare, Waukesha, WI 53188 USA; 3 Department of Medical Radiation Physics and Nuclear Medicine, Karolinska University Hospital, 171 76 Stockholm, Sweden; 4 Department of Clinical Neuroscience, Karolinska Institutet and Department of Neuroradiology, 171 74 Stockholm, Sweden; 5 Anatomical Research Training and Education, Vrije Universiteit Brussel, 1090 Brussels, Belgium

**Keywords:** Phantoms (imaging), Semiconductors, Stents, Tomography (x-ray computed), Vascular diseases

## Abstract

**Background:**

In this study, stent appearance in a novel silicon-based photon-counting computed tomography (Si-PCCT) prototype was compared with a conventional energy-integrating detector CT (EIDCT) system.

**Methods:**

An *ex vivo* phantom was created, consisting of a 2% agar-water mixture, in which human-resected and stented arteries were individually embedded. Using similar technique parameters, helical scan data was acquired using a novel prototype Si-PCCT and a conventional EIDCT system at a volumetric CT dose index (CTDI_vol_) of 9 mGy. Reconstructions were made at 50^2^ and 150^2^ mm^2^ field-of-views (FOVs) using a bone kernel and adaptive statistical iterative reconstruction with 0% blending. Using a 5-point Likert scale, reader evaluations were performed on stent appearance, blooming and inter-stent visibility. Quantitative image analysis was performed on stent diameter accuracy, blooming and inter-stent distinction. Qualitative and quantitative differences between Si-PCCT and EIDCT systems were tested with a Wilcoxon signed-rank test and a paired samples *t*-test, respectively. Inter- and intra-reader agreement was assessed using the intraclass correlation coefficient (ICC).

**Results:**

Qualitatively, Si-PCCT images were rated higher than EIDCT images at 150-mm FOV, based on stent appearance (*p* = 0.026) and blooming (*p* = 0.015), with a moderate inter- (ICC = 0.50) and intra-reader (ICC = 0.60) agreement. Quantitatively, Si-PCCT yielded more accurate diameter measurements (*p* = 0.001), reduced blooming (*p* < 0.001) and improved inter-stent distinction (*p* < 0.001). Similar trends were observed for the images reconstructed at 50-mm FOV.

**Conclusions:**

When compared to EIDCT, the improved spatial resolution of Si-PCCT yields enhanced stent appearance, more accurate diameter measurements, reduced blooming and improved inter-stent distinction.

**Key points:**

• This study evaluated stent appearance in a novel silicon-based photon-counting computed tomography (Si-PCCT) prototype.

• Compared to standard CT, Si-PCCT resulted in more accurate stent diameter measurements.

• Si-PCCT also reduced blooming artefacts and improved inter-stent visibility.

## Background

Since its introduction in the 1970s, computed tomography (CT) has been the subject of tremendous technical advances in both data acquisition and image reconstruction aspects. These vast technical advances have made CT a key player in non-invasive diagnostic imaging [1]. Recently, a new type of detection technique in CT has been introduced: photon-counting CT (PCCT). Opposed to conventional energy-integrating detector CT (EIDCT) systems, equipped with scintillator detectors, PCCT detector technology relies on the use of a different detector materials, *i.e.,* semiconductors [2]. Based on semiconductor detector physics and technology, PCCT is expected to provide an enhanced spatial resolution through pixel size reduction [3, 4].

Research into semiconductor detector materials for PCCT has mainly been focused on cadmium telluride (CdTe) and silicon (Si). The most common semiconductor today is CdTe, whereas research on silicon-based photon-counting CT (Si-PCCT) is rather limited as only recently a novel Si-PCCT prototype system has been introduced [5–8].

In vascular imaging, the use of CT has a long history in disease diagnosis and is also used for non-invasive follow-up of stented patients [9]. Follow-up imaging using CT is recommended as complications may arise, compromising patient health and requiring monitoring and revascularization [10, 11]. Complications after vascular stent implantation may include in-stent stenosis, aneurysm formation, in-stent thrombosis and stent fractures or displacement [12–14]. Detection of possible complications remains challenging with state-of-the-art EIDCT systems as these are vulnerable to the effects of blooming and metal artefacts from the constituent metal, photon starvation and partial volume effects. These artefacts may lead to overestimation of stent diameter and underestimation of vessel size, highlighting the need for a more accurate follow-up examination tool [12, 15].

With the rise of PCCT, it is anticipated that this new detector technology has the potential to overcome the current limitations associated with EIDCT as the extent of PCCTs’ superior spatial resolution has already proven its potential in other diagnostic imaging applications [2, 16]. In vascular stent follow-up, PCCTs’ increased resolution has also been evaluated, demonstrating beneficial capabilities in this area [17]. However, preceding studies were performed using CdTe-PCCT, whereas reports on Si-PCCT remain scarce. Therefore, the purpose of this study is to evaluate stent appearance in an anatomical *ex vivo* phantom by means of a head-to-head comparison between a novel Si-PCCT prototype and a conventional EIDCT system.

## Methods

### ***Ex vivo*** phantom

**Fig. 1:**
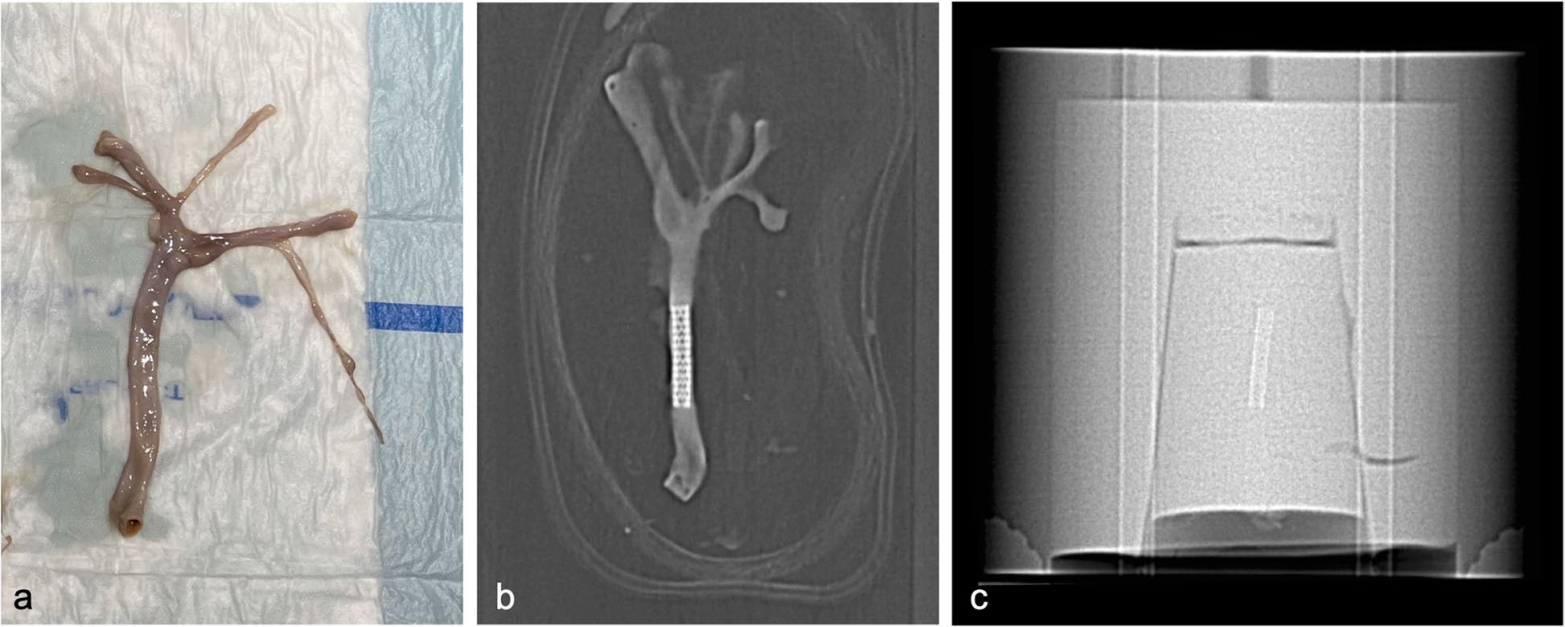
Resected carotid artery prior to stent placement (**a**), topogram of the stented carotid artery (**b**), and topogram of the *ex vivo* phantom containing the stented carotid artery (**c**)

Ethical approval was obtained for the use of three arteries (carotid, femoral and iliac), which were resected from a human cadaver at our university anatomy lab (Fig. 1a). After resection, an interventional radiologist introduced four stents (Atrium Advanta V12 over-the-wire covered stents, Atrium Medical Corporation, New Hampshire, USA), made from the same material (stainless steel encapsulated with polytetrafluoroethylene), using a delivery system consisting of an over-the-wire catheter with a 0.035-inch guidewire, followed by inflation to a nominal pressure of 811 kPa. One stent was placed in each specimen (Fig. 1b), except for the femoral artery in which two intertwined stents were placed for the evaluation of inter-stent visibility and distinction. The stented arteries were filled with an iodinated contrast material solution (Iomeron 350 mixed with 0.9% saline; Bracco Imaging GmbH, Konstanz, Germany) diluted to a concentration of 15 mg/mL. Next, the stented arteries were individually embedded at a central position in a 20-cm diameter cylindrical phantom, filled with a 2% water-agar mixture to mimic soft tissue (Fig. 1c).

### CT data acquisition and reconstruction

Data acquisition was performed using a prototype Si-PCCT system (GE Healthcare, Milwaukee, WI, USA), which was equipped with a silicon semiconductor photon-counting detector. EIDCT data acquisition was performed using a clinical CT system (Revolution CT, GE Healthcare, Milwaukee, USA), equipped with an energy-integrating detector. On both systems, the phantom was scanned with a helical acquisition protocol at comparable technical settings, achieving a volumetric CT dose index (CTDI_vol_) of approximately 9 mGy (Table 1).

**Table 1:** System parameters of the prototype silicon-based photon-counting computed tomography (Si-PCCT) and clinical energy-integrating detector CT (EIDCT) systems

	Si-PCCT	EIDCT
Detector material	Silicon semiconductor	Gemstone scintillator
CTDI_vol_ (32 cm)	8.9 mGy	9 mGy
Scan mode	Helical	Helical
Pitch	0.99	0.98
Bowtie filter	Medium	Head (medium body)
Tube voltage	120 kVp	120 kVp
Tube current	255 mA	245 mA
Tube rotation	0.5 s	0.5 s
Focal spot size	0.6 mm	1.2 mm
*z*-coverage	40 mm	40 mm
Matrix	1024 × 1024	512 × 512
Field-of-view (FOV)	150 × 150 mm (150-mm FOV) 50 × 50 mm (50-mm FOV)	150 × 150 mm (150-mm FOV) 50 × 50 mm (50-mm FOV)
Pixel size	0.146 mm (150-mm FOV) 0.049 mm (50-mm FOV)	0.293 mm (150-mm FOV) 0.098 mm (50-mmFOV)
Slice thickness	0.42 mm	0.63 mm
Kernel	Bone + ASIR0	Bone + ASIR0

*ASIR0* Adaptive statistical iterative reconstruction with 0% blending, *CTDI*_*vol*_ Volumetric computed tomography dose index, *EIDCT* Energy-integrating computed tomography, *PCCT* Photon-counting computed tomography, *FOV* Field-of-view

Si-PCCT image data was reconstructed with a slice thickness of 0.42 mm, a field-of-view (FOV) of 150 × 150 mm^2^ (150-mm FOV) and 50 × 50 mm^2^ (50-mm FOV) and an image matrix of 1024^2^, which resulted in a pixel size of 0.146^2^ mm^2^ and 0.049^2^ mm^2^, respectively. EIDCT image data was reconstructed with a slice thickness of 0.63 mm, the same two FOV and an image matrix of 512^2^, which resulted in a pixel size of 0.293 × 0.293 mm^2^ and 0.098 × 0.098 mm^2^, respectively. Both image datasets were reconstructed with the same clinical bone kernel using adaptive statistical iterative reconstruction with 0% blending.

### Qualitative stent evaluation

Two radiologists, having a vascular imaging experience of 30 and 20 years, independently assessed the Si-PCCT and EIDCT datasets on a radiological workstation equipped with medical-grade displays (Barco MXRT 4700, Barco, Kortrijk, Belgium). During each evaluation, the readers were presented with a total of 16 images (8 Si-PCCT and 8 EIDCT) in a random order while being blinded to imaging mode and sample. Stent appearance, blooming and inter-stent visibility were assessed qualitatively using a 5-point Likert score (Table 2). The evaluation was performed on axial, sagittal and coronal slices at a fixed window with (WW) of 3,700 Hounsfield units (HU) and window centre/level (WL) of 1,350 HU. Three-dimensional maximum intensity projection (3DMIP) images were evaluated using the same WW and WL, whereas three-dimensional volume-rendered (3DVR) images were evaluated at a fixed WW and WL of 100 HU and 1,050 HU, respectively. The readers were free to zoom and roam through the image volumes, the 3DVR and 3DMIP images in any plane using an image viewing software (RadiAnt Dicom Viewer, 2022.11; Medixant, Poznan, Poland).

**Table 2:** Five-point Likert scores for stent evaluation on stent appearance, blooming and inter-stent visibility

Score	Stent appearance	Blooming	Inter-stent visibility
5	Very clear	No blooming	Very clearly distinguishable
4	Clear stent	Minimal	Clearly distinguishable
3	Moderate	Moderate	Moderately distinguishable
2	Poor	Marked	Minimally distinguishable
1	Insufficient	Severe	Impossible to distinguish

### Quantitative stent evaluation

**Fig. 2:**
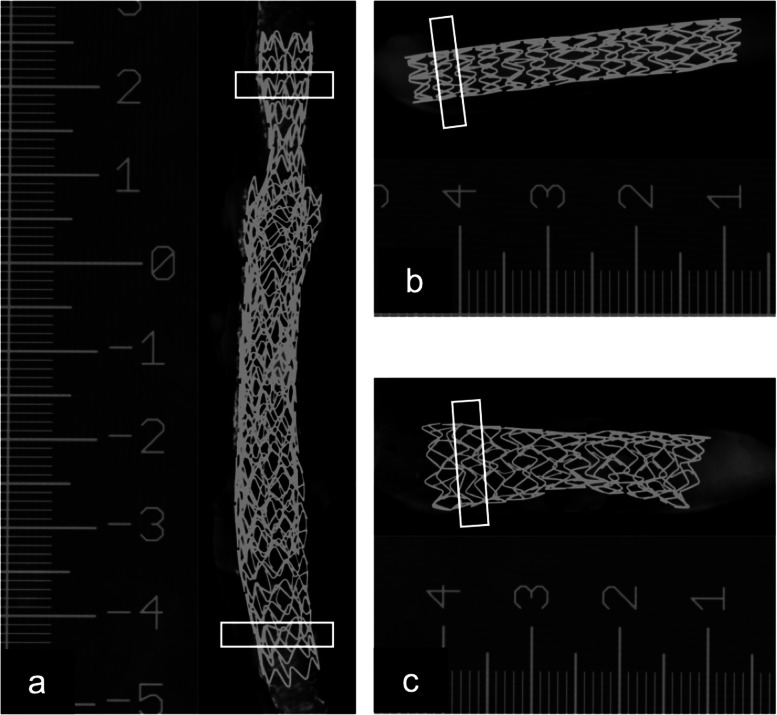
True stent diameter measurements using spot mammography of the stents in the double-stented femoral artery (**a**), carotid artery (**b**) and iliac artery (**c**). Boxes indicate the regions used for quantitative image analysis. A ruler as scale was included while acquiring the images

Quantitative assessment of stent appearance was performed using diameter measurement accuracy, blooming artefacts and inter-stent distinction. Prior to image analysis, measurements of the true stent diameter were made using spot mammography (Senograph Pristina, General Electric Healthcare, Brussels, Belgium) using a calliper tool in a picture archiving and communications system (PhilipsVuePACS, Brussels, Belgium) (Fig. 2). These measurements served as ground truth to assess the measured diameter using the Si-PCCT and EIDCT systems: carotid (*d* = 5.5 mm), femoral (*d*1 = 7 mm, *d*2 = 5.8 mm) and iliac (*d* = 8.5 mm).

**Fig. 3:**
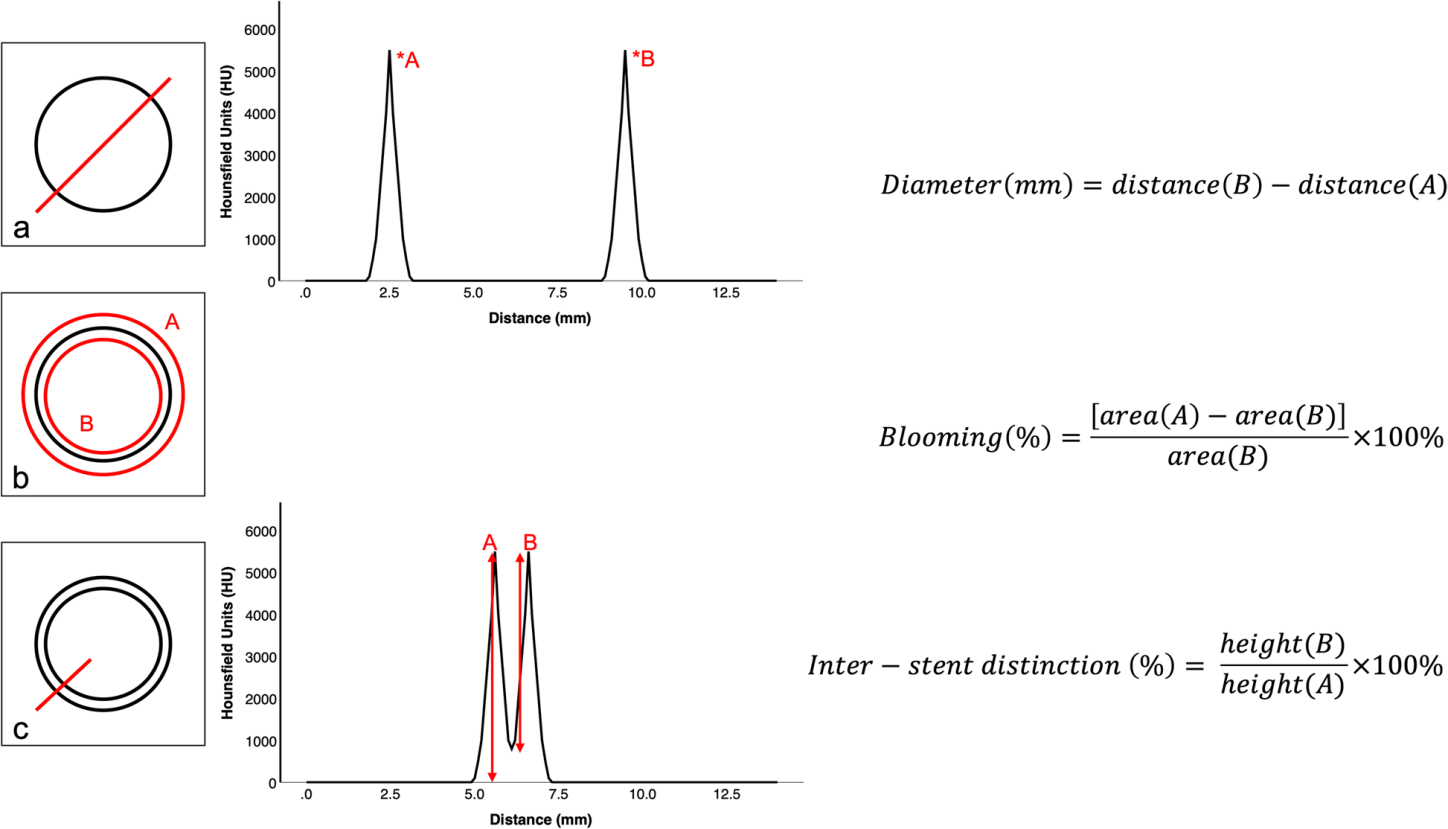
Quantitative image analysis methodologies and calculations for assessment of diameter measurement accuracy (**a**), blooming (**b**), and inter-stent distinction (**c**); *x*- and *y*-axes are representative for the average observed density (HU) and distances (mm)

On the CT data, average stent diameters (mm) were measured, using a peak-to-peak method on axial slices by plotting HU over distance (mm), *i.e.,* HU profile, using a lining and calliper tool (Fig. 3a). The measured stent diameters were compared with the true stent diameter to compute the mean error value. Blooming was calculated as the relative difference between the inner and outer area (mm^2^), expressed as a percentage, based on a method described in a previous study [18]. Average inner and outer areas were measured using a circular region-of-interest drawing tool (Fig. 3b). For the specimen with the two stents, the average inter-stent distinction was defined as the relative peak-to-trough distance, after plotting HU over distance (mm) for the assessment of average minimum (HU), peak (HU) and trough (HU) (Fig. 3c). For each of the above measurements, three locations were considered for each stent at the same location (for Si-PCCT and EIDCT) and at a fixed WW of 1,350 HU and WL of 3,700 HU using an open-source imaging processing software (ImageJ, National Institutes of Health, USA).

### Statistical analysis

Continuous variables were expressed as means $\pm$ standard deviations (SD), and categorical variables as a median score with Q1–Q3 interquartile range (IQR) on a 5-point scale. The distribution of the continuous variables was tested and confirmed for normality by use of the Shapiro–Wilk test. Differences in qualitative scores on stent evaluation between Si-PCCT and EIDCT were tested using a Wilcoxon signed rank test while inferring a two-tailed *p*-value < 0.05 to indicate significance. Inter- and intra-reader agreement for qualitative imaging parameters (stent appearance, blooming, inter-stent visibility) was assessed using an intraclass correlation coefficient (ICC). ICC estimates were calculated based on a mean rating (2 readers), consistency agreement and a two-way mixed effects model. Interpretation of ICC follows the characterization according to guidelines on ICC by Koo and Mae [19]: poor reliability (< 0.5), moderate reliability (0.5–0.75), good reliability (0.75–0.9) and excellent reliability (> 0.9).

For the three stents together, mean differences in quantitative image parameters (diameter measurement accuracy, blooming, inter-stent distinction) between Si-PCCT and EIDCT were tested using a paired samples *t*-test while inferring a two-tailed with *p*-value < 0.05 to indicate significance. All statistical analyses were conducted using a commercially available statistics software (SPSS, release 28; IBM, Chicago, IL, USA).

## Results

### Qualitative stent evaluation

**Fig. 4:**
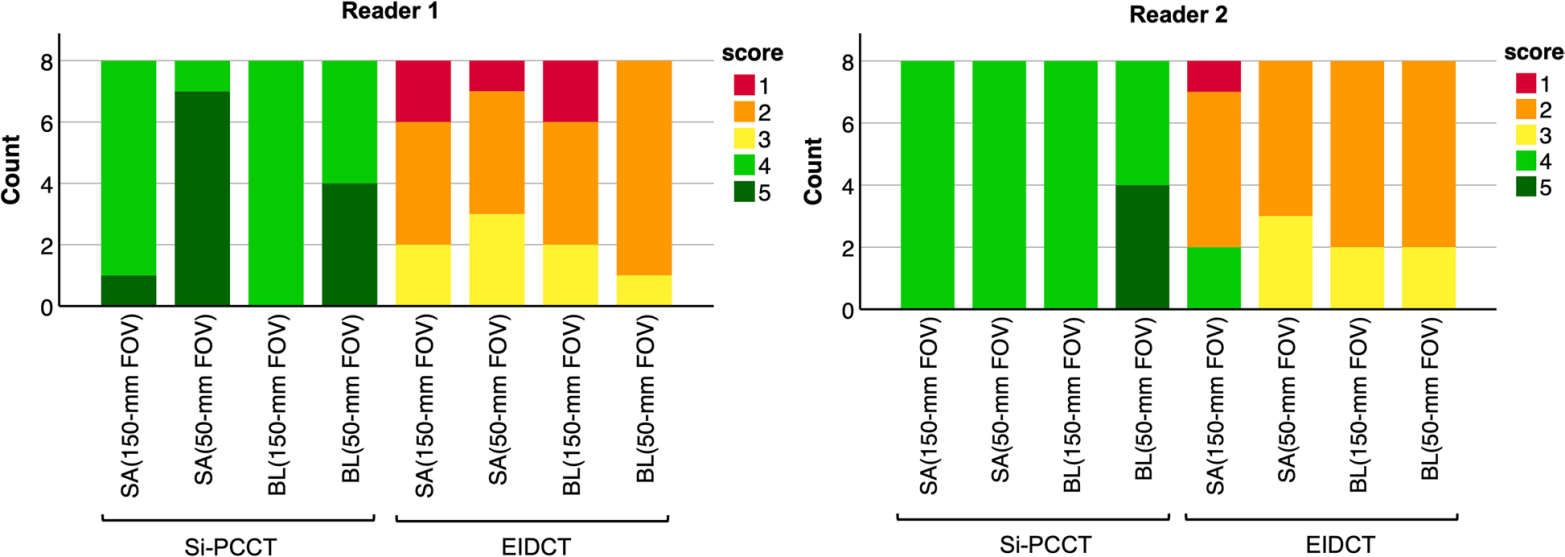
Reader evaluation results (based on a 5-point Likert scale) on stent appearance (SA) and blooming (BL) at 150-mm and 50-mm field-of-view (FOV) for silicon-based photon-counting CT (Si-PCCT) and energy-integrating detector CT (EIDCT) images. Stent appearance was found to be insufficient (score 1) for 12.5% (4 out of 32) of the EIDCT images. Two EIDCT images (out of 32) were perceived as containing severe blooming artefacts (score 1)

**Fig. 5:**
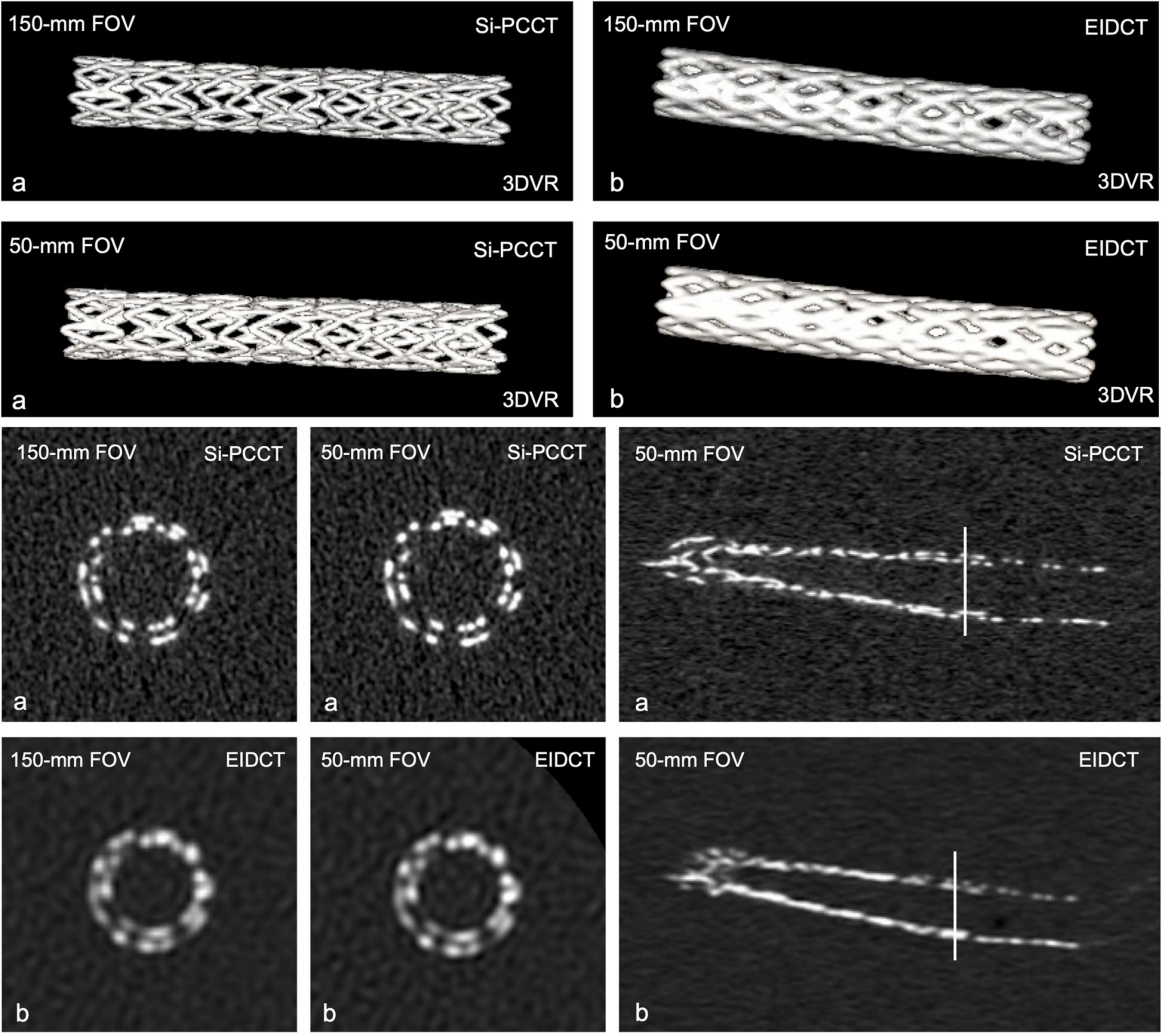
Three-dimensional volume-rendered (3DVR) images, axial and coronal slices at 150-mm and 50-mm field-of-view (FOV) for silicon-based photon-counting CT (Si-PCCT) (**a**) and energy-integrating detector CT (EIDCT) (**b**). Qualitative stent appearance was found to be insufficient (score 1) for 12.5% of the EIDCT images (both FOVs) due to impaired visibility of stent struts and impaired stent delineation, as represented in the 3DVR images

At both FOVs, average stent appearance was perceived on average significantly better for Si-PCCT (150-mm FOV: median 4, IQR 4–4; 50-mm FOV: 4, 4–5) compared to EIDCT (2, 2–2.75 and 2, 2–3, respectively), *p* = 0.026 (150-mm FOV) and *p* = 0.010 (50-mm FOV). For Si-PCCT, all images were considered sufficient (score > 1) whereas 12.5% (4 out of 32 EIDCT images) were assessed as insufficient due to impaired visibility of stent struts and stent delineation (Figs. 4 and 5).

With respect to the appearance of blooming artefacts, average Likert ratings at both FOVs were found to be significantly improved at both FOVs for Si-PCCT (150-mm FOV: median 4, IQR 4–4; 50-mm FOV: 4.5, 4–5) compared to EIDCT (2, 2–2.75 and 2, 2–2), *p* = 0.015 (150-mm FOV) and *p* = 0.010 (50-mm FOV). Similar to stent appearance, no Si-PCCT images were graded with a score of 1 (severe blooming), whereas two EIDCT images (out of 32) were perceived as containing severe blooming artefacts (Figs. 4 and 5).

For both FOVs, average inter-stent visibility reader scores were higher for Si-PCCT (150-mm FOV: median 3.5, IQR 3–4.75; 50-mm FOV: 4.5, 4–5) compared to EIDCT (2, 1.25–2 and 2, 2–2.75). However, these differences were not found to be statistically significant (150-mm FOV: *p* = 0.180; 50-mm FOV: *p* = 0.157). Stents in one EIDCT image were rated as being impossible to distinguish, whereas all scores relating to inter-stent visibility in Si-PCCT images were rated as distinguishable (Fig. 5). Inter- and intra-reader agreement across all evaluations was found to be moderate (ICC 0.50 and 0.60, respectively).

### Quantitative stent evaluation

For the Si-PCCT images at 150 mm FOV, the mean error towards the true diameter was significantly lower (0.17 mm ± 0.16 mm) compared to the 150 mm FOV EIDCT images (0.59 mm ± 0.26 mm) (*p* = 0.001). At 50-mm FOV, the mean error towards the true diameter did not differ significantly between the two systems (Si-PCCT 0.21 mm ± 0.26 mm *versus* EIDCT 0.26 mm ± 0.19 mm; *p* = 0.242) (Table 3).

**Table 3:** Quantitative analysis results on diameter measurement accuracy, blooming and inter-stent distinction

		Mean error towards true diameter	Blooming	Inter-stent distinction
150-mm FOV	Si-PCCT	0.17 mm (± 0.16 mm)*	18.3% (± 2.6%)**	80.7% (± 7.6%)**
	EIDCT	0.59 mm (± 0.26 mm)*	32.4% (± 4.6%)**	49% (± 15.8%)**
50-mm FOV	Si-PCCT	0.21 mm (± 0.26 mm)	15.3% (± 3.3%)**	84% (± 11.8%)*
	EIDCT	0.26 mm (± 0.19 mm)	28.1% (± 3.7%)**	60.9% (± 8.5%)*

*EIDCT* Energy-integrating computed tomography, *FOV* Field-of-view, *PCCT* Photon-counting computed tomography

^*^p < 0.01, **p < 0.001 (two-tailed) when comparing Si-PCCT to EIDCT

**Fig. 6:**
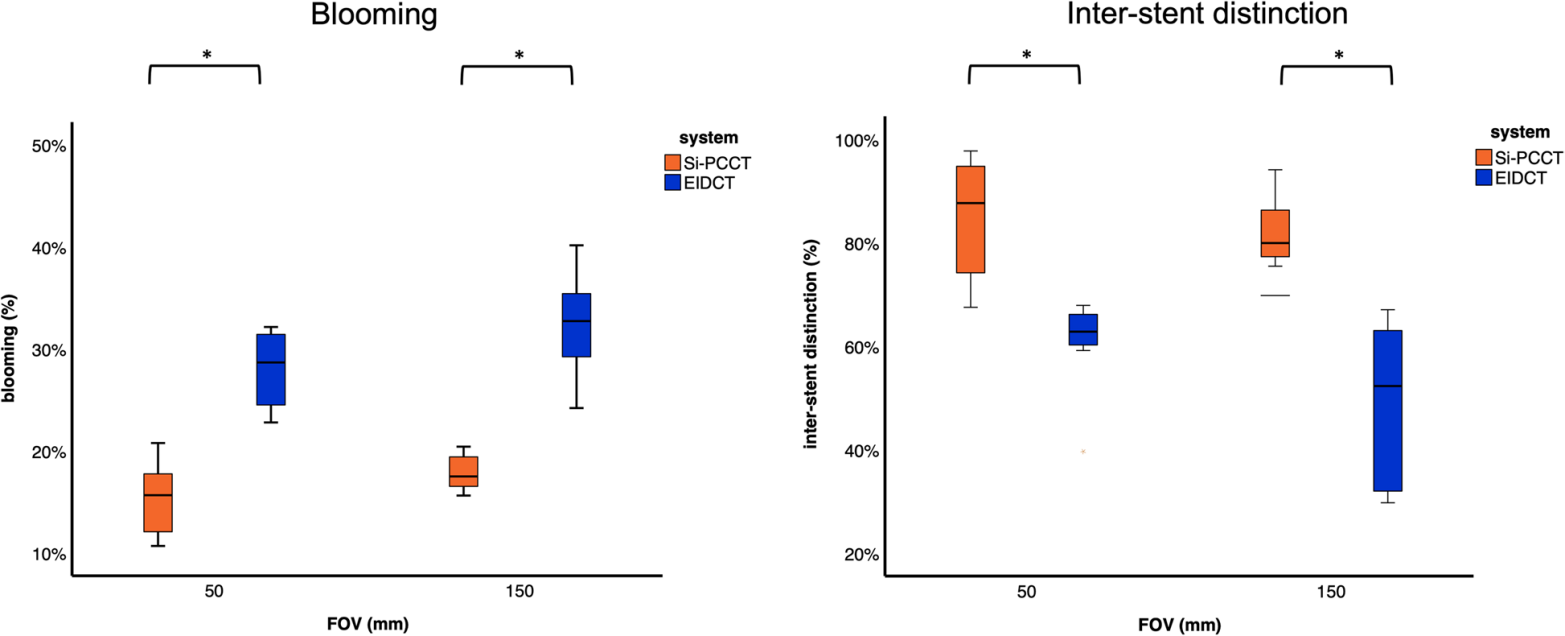
Box plots representing average percentages of (left) blooming (%) and (right) inter-stent distinction (%) for silicon-based photon-counting CT (Si-PCCT) and energy-integrating detector CT (EIDCT) at 150-mm and 50-mm field-of-view (FOV)

For images at both FOVs, the average blooming percentage was found to be significantly lower for Si-PCCT (150-mm FOV: 18.3% ± 2.6%; 50-mm FOV: 15.3% ± 3.3%) compared to EIDCT (150-mm FOV: 32.4% ± 4.6%; 50-mm FOV 28.1% ± 3.7%), indicating reduced blooming artefacts on Si-PCCT images, *p* < 0.001 (Table 3, Fig. 6a).

Likewise, the average inter-stent distinction was measured to be significantly higher for Si-PCCT at both FOVs (150-mm FOV: 80.7% ± 7.6%; 50-mm FOV: 84% ± 11.8%) when compared to EIDCT (150-mm FOV: 49% ± 15.8%; 50-mm FOV: 60.9% ± 8.5%), indicating a higher extent of stent distinction in Si-PCCT, *p* < 0.001 (150-mm FOV) and *p* = 0.003 (50-mm FOV) (Table 3, Fig. 6b). These quantitative results reinforce the qualitative findings from the reader study.

## Discussion

Our study evaluated the degree of improved spatial resolution in a prototype Si-PCCT system using an *ex vivo* phantom consisting of stented arteries. Qualitative and quantitative metrics of stent appearance, image artefacts due to blooming and the ability to distinguish intertwined stents were compared between an investigational Si-PCCT and a conventional EIDCT system. Potential advantages of Si-PCCT in the spectral space, including material identification, discrimination and characterization, were not investigated.

The findings of this study clearly demonstrate both qualitative and quantitative improvements in spatial resolution of a Si-PCCT system over an EIDCT system. Improved spatial resolution in a CdTe-PCCT has already been investigated and confirmed in various simulations and experimental studies using dedicated phantoms [20–23]. Also in Si-PCCT, spatial resolution was reported to be substantially higher compared to EIDCT [24].

In the present study, the enhanced spatial resolution of a prototype Si-PCCT system allowed a more precise rendition of high-contrast boundaries, resulting up to 80% more accurate stent diameter measurements. These findings are in line with a recently published study by Rajagopal et al. [20], where similar trends in measurement accuracy (40% more accurate measurement of diameter in CdTe-PCCT compared to EIDCT) were found using a similar phantom set-up. Blooming artefacts of the individual stent struts were also reduced due to Si-PCCTs enhanced resolution. Using EIDCT, blooming artefacts might cause reduced in-stent lumen visibility which remains a challenging issue. A study by Bratke et al. [25] even demonstrated the inability of EIDCT systems to accurately evaluate in-stent stenosis due to severe blooming, whereas CdTe-PCCT was able to accurately allow evaluation of in-stent lumen. On Si-PCCT images, we could demonstrate a significant blooming reduction of approximately 55%. In a study of Sigovan et al. [22], similar trends were observed as stent struts could be measured with a 30% increase of accuracy in CdTe-PCCT. More accurate stent strut diameter measurements are due to reduced blooming of the struts, which confirms findings in the present study. In the qualitative evaluation, Si-PCCT stent images were also preferred in terms of blooming. Lastly, we were able to better depict intertwined stents on Si-PCCT images, having an inter-stent distinction up to 84%, whereas conventional EIDCT had a limited distinction of 61% at the smallest FOV. Qualitative evaluation also confirms these results as higher average scores were obtained for Si-PCCT stent images on inter-stent visibility. The ability to distinguish two intertwined stents strongly depends on the precise rendition of high-contrast boundaries and blooming. Severe blooming limits the capability of inter-stent distinction as it causes the stents to appear as one.

Our study has several limitations. We evaluated an *ex vivo* vascular phantom, yet, this experimental set-up allowed us to acquire repeated and identical phantom images of human samples on two systems with different detector technologies which could be used in a direct comparison between the two types of CT systems at a similar radiation dose, which is not possible in a clinical patient study. By using an *ex vivo* anatomical phantom, we did not consider motion artefacts. In future research, *ex vivo* phantoms in which the pulsatile vessel motion can be simulated could be considered to include motion artefacts [26].

Our study also only covered spatial resolution characteristics in terms of stent appearance. No other evaluations on noise or plaque identification, iodine enhancement and lumen visibility were considered. We did not evaluate the impact of different reconstruction kernels. However, this could be a valuable approach to further improve qualitative and quantitative evaluations of stent appearance [27]. Lastly, being a first hand-on experience study, only a limited number of samples were considered. However, this initial study enables future projects that will investigate additional clinical phantom-based imaging tasks, which may benefit from the improved spatial resolution and spectral imaging capabilities enabled by Si-PCCT.

In conclusion, when compared to current state-of-the-art clinical EIDCT systems, Si-PCCT offers an improved spatial resolution as stent appearance was substantially improved. Both qualitatively and quantitatively, the improved spatial resolution of Si-PCCT yielded an enhanced stent appearance and more accurate diameter measurements and allowed two intertwined stents to be clearly distinguishable.

## Data Availability

The datasets used and analyzed during the current study are available from the corresponding author on reasonable request.

## Abbreviations

3DMIP: Three-dimensional maximum intensity projection
3DVR: Three-dimensional volume rendered
CdTe: Cadmium telluride
CdTe-PCCT: Cadmium telluride-based photon-counting computed tomography
CT: Computed tomography
CTDI_vol_: Computed tomography dose index volume
EIDCT: Energy-integrating computed tomography
FOV: Field-of-view
HU: Hounsfield unit
ICC: Intraclass correlation coefficient
PCCT: Photon-counting computed tomography
Si-PCCT: Silicon-based photon-counting CT
WL: Window level (or window centre)
WW: Window width
